# Down-expression of klotho in canine mammary gland tumors and its prognostic significance

**DOI:** 10.1371/journal.pone.0265248

**Published:** 2022-06-06

**Authors:** Heaji Chung, Sungin Lee, Geon A. Kim, Wan Hee Kim

**Affiliations:** 1 Department of Veterinary Clinical Sciences, College of Veterinary Medicine and Research Institute for Veterinary Science, Seoul National University, Seoul, Republic of Korea; 2 Department of Veterinary Surgery, College of Veterinary Medicine, Chungbuk National University, Cheongju, Republic of Korea; 3 Department of Clinical Pathology, University of Health Science, Eulji University, Uijeongbu, Republic of Korea; Colorado State University, UNITED STATES

## Abstract

Since the discovery of klotho as an anti-aging gene, its association with tumors has been studied. Several previous studies have reported the down-expression of klotho in various human cancers, and much of its mechanism has been revealed. Nonetheless, the significance of klotho in canine mammary gland tumors is not yet known. This study aimed to determine whether klotho is expressed within normal canine mammary glands and whether the expression changes in benign and malignant tumors. Using immunohistochemistry, the experiment was conducted on eight normal canine mammary gland tissues and 55 mammary gland tumor samples. Additionally, the correlation between the Ki-67 proliferation index and clinicopathological features, such as age, tumor size, tumor grade, histologic type, and metastasis, was evaluated. All eight normal mammary gland tissues showed immunohistochemistry expression of klotho, and the expression significantly decreased as malignancy increased. Among the samples, 11% (3/28) of benign tumors and 26% (7/27) of malignant tumors showed negative klotho expression. Furthermore, higher Ki-67 expression, higher grades, and metastasis were confirmed to be associated with the negative klotho expression. Analysis of the survival curve for dogs with malignant tumors revealed that negative klotho expression was significantly associated with poor overall survival and disease-free survival. These results indicate that klotho is expressed in normal canine mammary glands and that negative klotho expression in canine mammary gland tumors is positively correlated with poor prognosis.

## Introduction

Klotho was first discovered as an anti-aging gene by Kuro-o et al. [1]. Mice with the mutation of the klotho gene showed features of aging, such as short life span, infertility, and skin atrophy, whereas klotho-overexpressed mice had increased life span [1, 2]. Since then, several studies have been conducted to elucidate the mechanism of klotho action on aging. Klotho encodes a transmembrane protein, and the extracellular domain of this protein can be secreted externally. Klotho is expressed in two different forms with distinct functions—namely, the membrane form and the circulating form (soluble form). The membrane form functions as the co-receptor for fibroblast growth factor 23 (FGF-23). Defects of either FGF-23 or klotho cause phosphate retention, which is known to accelerate aging [3, 4]. Circulating klotho represses insulin and insulin-like growth factor 1 (IGF-1) signaling, which results in a resistance increase to oxidative stress [2, 5].

As cancer is part of the aging process, it is natural for klotho to play a tumor suppressor role. Many studies have found decreased expression of klotho protein in malignant tumors compared with that in normal tissues. It has been observed using immunohistochemistry in the human breast [6], gastric [7], colorectal [8], oesophageal [9], pancreatic [10], renal [11], hepatocellular [12] and ovarian cancers [13]. Forced expression of klotho in breast cancer cells reduced proliferation, whereas klotho silencing resulted in increased proliferation [6]. Klotho can suppress cancer by inhibiting IGF-1 signaling, which is known to be upregulated in cancer cells and promotes cancer growth and metastasis [6, 14]. In addition, klotho has been found to inhibit tumor growth by acting on the FGF, phosphatidylinositol 3-kinase/Akt, WNT, transforming growth factor β, and unfolded protein response pathways. To this end, research has recently been underway to increase klotho expression in various ways for the application of klotho as a new therapeutic agent for cancer [15].

Canine mammary gland tumors are the most common tumors in intact females, and up to 53.3% of intact females are found to have malignant mammary gland tumors [16]. It can be benign or malignant and can progress from benign to malignant lesions [17]. The prognosis of canine mammary gland tumors depends on the histologic tumor type, tumor size, and the presence of metastasis [18]. Because the level of malignancy varies, and the tumor’s behavior patterns vary accordingly, evaluation of prognosis is important. The main treatment option is surgical removal, which is considered the single most effective treatment to achieve local tumor control, excluding inflammatory carcinomas or tumors with distant metastases [19]. Adjuvant therapies such as radiation, hormonal therapy, and chemotherapy are not routinely used in veterinary medicine, and their effects have not been revealed [20].

Earlier research has observed changes in klotho expression in various human tumors and their mechanism of action. Klotho has also been studied for its potential as a prognostic factor [9, 13, 21] and therapeutic target in cancer [7, 22]. To the best of our knowledge, the relationship between klotho expression and canine mammary gland tumors has not been studied. In this study, klotho expression in canine mammary glands was investigated and its expression in normal canine breast, benign tumor, and malignant tumor was compared by immunohistochemistry. In addition, by identifying its association with several clinicopathological factors and klotho expression, we assessed the potential of klotho as a prognostic factor. This study can lay the foundation for further research on the development of therapeutic agents for canine mammary gland tumors.

## Materials and methods

### Tissue samples

Normal mammary gland tissues were obtained from eight 2-year-old intact female beagles housed in the Department of Veterinary Surgery, College of Veterinary Medicine at Seoul National University. Physical examination, serum biochemistry, complete blood count, radiography, and abdominal ultrasonography confirmed that all dogs were healthy. The dogs had never been pregnant, and a vaginal smear was used to confirm that the dogs were in the anestrus period. The mammary gland tissue samples were collected after the euthanasia of the subjects of another experiment (SNU-181214-3), which is not expected to affect the results of this study. All samples were fixed in 10% neutral buffered formalin at room temperature for 48 h. After embedding, paraffin blocks were cut into 3-μm sections and stained with hematoxylin and eosin to histologically confirm normal mammary glands.

A total of 28 benign and 27 malignant mammary gland tumors were collected from dogs that underwent surgery between January 2017 and December 2020 at Seoul National University Medical Teaching Hospital. The owners of mammary gland tumour dogs were informed about the procedure and purpose of this study, and informed consent was obtained. Tissues diagnosed as mammary gland tumors based on the histopathological examination of the Veterinary Pathology Laboratory of Seoul National University or IDEXX Laboratories were included. A portion of the resected tumor tissue was collected immediately after surgery. Clinical data about patients, tumor size, and metastasis were also collected. Every tissue was fixed, sectioned, and stained in the same way as for the normal mammary glands. Malignant tumors were graded according to the diagnostic criteria of Goldschmidt et al. [23]. As recommended by Sorenmo [24], if more than one mammary gland tumor was identified in a dog, a tumor with more aggressive pathological features was selected. Animals with other malignant tumors detected as a result of various screening tests before surgery were excluded from the sample patients. The protocol and procedures employed were ethically reviewed and approved by the Institutional Animal Care and Used Committee of Seoul National University (SNU-210305-3).

### Immunohistochemistry

Paraffin-embedded tissues were cut into 3-μm-thick sections. These sections were deparaffinized with xylene three times for 5 min each and rehydrated in graded alcohol (100%, 95%, 70%, and distilled water for 5 min each). Antigen retrieval was carried out with 10 mM citrate acid buffer (pH 6) for 10 min at 97°C, and endogenous peroxidase activity was quenched by incubation with 3% H_2_O_2_ for 10 min. The sections were treated with 1% bovine serum albumin (Sigma-Aldrich, St Louis, MO, USA) for 1 h to block nonspecific binding. The sections were incubated for 1 h at 37°C with rabbit anti-klotho polyclonal antibody (1:200, LS-B6625; LSBio, Seattle, WA, USA) and rabbit anti-Ki-67 polyclonal antibody (1:500, PA5-19462; Invitrogen Ltd., Paisley, UK). To serve as a negative control, the sections were incubated with rabbit IgG isotype control (1:200, 02–6102; Invitrogen Ltd., Paisley, UK). The sections were incubated with secondary antibody (Envision + system HRP Labeled Polymer anti-rabbit; Dako, Carpinteria, CA, USA) for 1 h at room temperature. The slides were treated with DAKO liquid with 3,3-diaminobenzidine tetrahydrochloride diaminobenzidine substrate chromogen system (DAKO) for 1 min. The C57BL/6J mouse brain tissue was used as a positive control. The slides were counterstained with Mayer’s hematoxylin and washed with phosphate-buffered saline. They were scanned using a digital slide scanner (Pannoramic 250 Flash III; 3DHISTECH Ltd., Budapest, Hungary) and analyzed using a digital microscopy application (CaseViewer; 3DHISTECH Ltd.).

### Quantification of immunohistochemistry staining

The klotho expression was estimated semiquantitatively using the immunostaining score, calculated as the product of the proportion score and the intensity score. The scores were independently evaluated by two observers. The criterion for determining the presence of klotho staining was 10%, as referenced in two studies [25, 26]. The proportion score was determined as 0 (<10%), 1 (10–25%), 2 (26–50%), 3 (51–75%), or 4 (>75%) according to the percentages of positive staining areas in five representative high-power fields. The staining intensity was scored as 1 (weak), 2 (medium), or 3 (strong). Samples with a score of ≤4 were considered to have low expression, and samples with a score >4 were considered to have high expression. Ki-67 staining analysis was performed for nuclear positivity. All positively stained cells in five representative high-power fields containing at least 1000 cells were counted. A high Ki-67 index value was defined when the nuclear staining percentage was >15%. A >15% cut-off threshold was used based on a previous study that divided the low-and high-risk groups based on the Ki-67 intensity to represent the prognosis of canine mammary gland tumors [27].

### Follow-up data

All dogs underwent regular check-ups 2 weeks after surgery and every 3–6 months. Tumor recurrence and metastasis were evaluated by physical examination, thoracic radiography (three views), abdominal ultrasound, and fine-needle aspiration. If necessary, computed tomography scan, biopsy, and autopsy were performed.

### Statistical analysis

All statistical analyses were performed using SPSS (IBM Corp., Armonk, NY, USA). For progressive evaluation of tumor malignancy (normal mammary gland, benign tumor, and malignant tumor) and tumor grade (grades 1, 2, and 3) in association with klotho expression, a linear-by-linear association test was applied. The correlation between klotho staining and other clinicopathologic factors was analyzed using Fisher’s exact test and chi-square test. A Shapiro–Wilk test was used to examine the age variables for normality, and the Mann–Whitney U test was used to compare variables according to klotho expression. Kaplan–Meier survival curves were plotted and compared using the log-rank test. Disease-free survival was calculated from the date of primary surgical treatment to the time of detection of the first local recurrence or metastasis. Dogs with no metastasis or recurrence until the end of the study period or until death were censored. The dogs lost to follow-up were also censored. Overall survival was defined as the interval from primary surgical treatment to death from mammary gland cancer. The dogs that died from causes unrelated to mammary gland tumors, were lost to follow-up, or were alive until the end of the study period were censored. Statistical significance was set at P<0.05.

## Results

### Clinical information

The characteristics (median age, sex, breed) of the dogs included in this study as well as the histologic types of each tumor are presented in Table 1. The histologic types were according to the criteria by Goldschmidt et al. [23]. The major breeds were Maltese and Yorkshire terriers.

**Table 1:** Clinical parameters of the dogs included in this study.

	Normal mammary glands (n = 8)	Benign tumour (n = 28)	Malignant tumour (n = 27)
Median age (range) (years)	2	11 (6–16)	12 (7–15)
Sex (n)			
Spayed female	0	5	12
Female	8	23	14
Breed (n)	Beagle (8)	Maltese (10)	Maltese (5)
		Yorkshire terrier (8)	Toy poodle (5)
		Mixed (5)	Shih-tzu (4)
		Cocker spaniel (2)	Yorkshire terrier (3)
		Toy poodle (2)	Mixed (3)
		Chihuahua (1)	Cocker spaniel (2)
			Dachshund (2)
			Chihuahua (1)
			Jindo dog (1)
			Japanese spitz (1)
Histologic type (n)	−	Adenoma, simple (7)	Carcinoma, simple (13)
		Adenoma, complex (14)	Carcinoma, complex (9)
		Benign mixed tumour (7)	Carcinoma, mixed (4)
			Adenosquamous carcinoma (1)
Histologic grade (n)	−	−	Grade 1 (11)
	−	−	Grade 2 (8)
	−	−	Grade 3 (8)

### Klotho expression in mammary gland tissues and tumors

**Fig 1:**
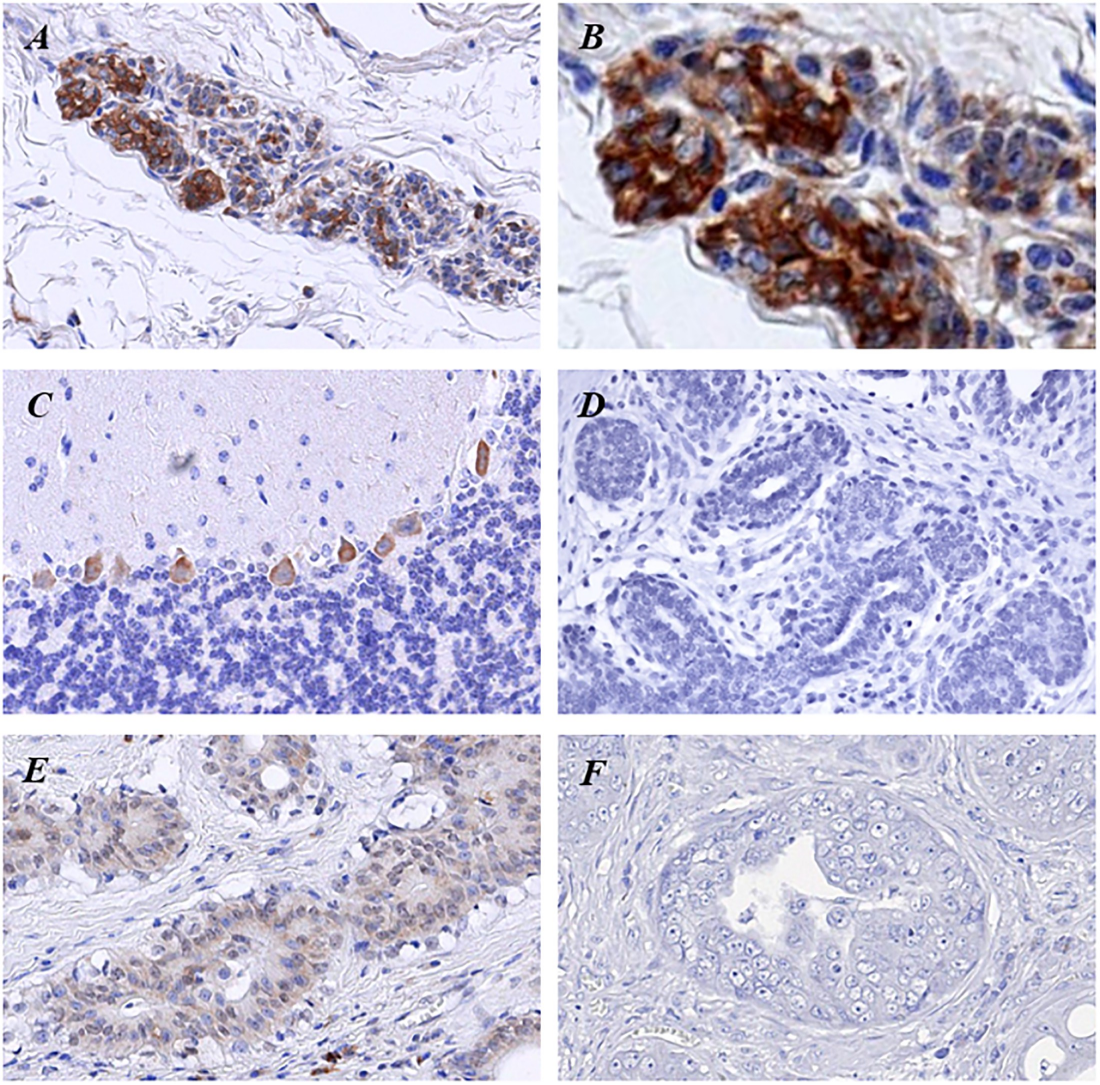
(A, B). Klotho immunohistochemistry in canine normal mammary glands. (C). In the positive control, klotho expression is observed in the Purkinje cells of the mouse brain cerebellum. (D). No specific staining is observed in the negative control. (E). Benign mammary gland tumor showing low klotho expression. (F). Malignant mammary gland tumor showing negative klotho expression. Original magnification: A, C, D, E, F, ×400; B, ×1000.

All eight canine mammary gland tissues showed positive immunoreactivity for klotho. Immunohistochemistry staining of klotho was mainly expressed in the cytoplasm of epithelial cells in the mammary glands (Fig 1A and 1B). Specific immunostaining of klotho was confirmed in the Purkinje cells in the cerebellum of the mouse brain as a positive control (Fig 1C) [28]. The negative control did not show klotho reaction (Fig 1D). As the degree of tissue malignancy increased, the staining intensity of klotho weakened (Fig 1E and 1F). In normal mammary glands, all tissues showed positive klotho expression, whereas, in benign and malignant tumors, 11% and 26% showed negative expression, respectively. This was statistically significant (Table 2). In addition, while 75% of normal mammary glands showed high immunoreactivity, the percentages of highly reactive tumors in the benign and malignant groups were 57% and 26%, respectively. The percentage of high klotho expression was significantly lower as the tumor malignancy increased (Table 2).

**Table 2:** Klotho expression in normal mammary glands, benign mammary gland tumours, and malignant mammary gland tumours.

Klotho expression n (%)	Normal mammary glands (n = 8)	Benign tumour (n = 28)	Malignant tumour (n = 27)	P value
Negative	0 (0)	3 (11)	7 (26)	P = 0.046* for negative vs. positive
Positive				
Low	2 (25)	9 (32)	13 (48)	P = 0.028* for low vs. high
High	6 (75)	16 (57)	7 (26)	

*P<0.05, indicating a statistically significant linear association with tumour malignancy and klotho expression level

### Correlation of clinicopathological factors and klotho expression

Table 3 shows the association between the clinicopathological variables and the presence of klotho expression. The negative and positive klotho expression groups had the same median age (11.5 years). Although the association with klotho expression was evaluated for tumors with sizes >3 cm in diameter, which are known to be associated with mammary tumor malignancy [17] it was not statistically significant. There was also no significant correlation between klotho expression and histologic diagnosis. For the malignant tumor group, grade, Ki-67 level, and the presence of metastasis during the follow-up period were significantly correlated with the absence of klotho expression.

**Table 3:** Clinicopathological variables associated with klotho expression.

	Number of tumours	Klotho expression		Overall P value
		Negative	Positive	
Median age (range) (years)		11.5 (10–15)	11.5 (6–16)	0.439
Tumour size (n)				
≤3 cm	34	5	29	
>3 cm	21	5	16	0.48
Benign tumour (n)				
Simple	7	1	6	
Complex	14	0	14	
Mixed	7	2	5	0.111
Malignant tumour (n)				
Simple	13	3	10	
Complex	9	4	5	
Mixed	4	0	4	
Adenosquamous	1	0	1	0.356
Histologic grade (n)				
Grade 1	11	1	10	
Grade 2	8	1	7	
Grade 3	8	5	3	0.013*
Ki-67 (n)				
≤15%	15	1	14	
>15%	12	6	6	0.024*
Metastasis (n)				
No	16	1	15	
Yes	11	6	5	0.009*

*P<0.05, indicating a statistically significant association between negative klotho expression and clinicopathological variables.

### Survival curve

**Fig 2:**
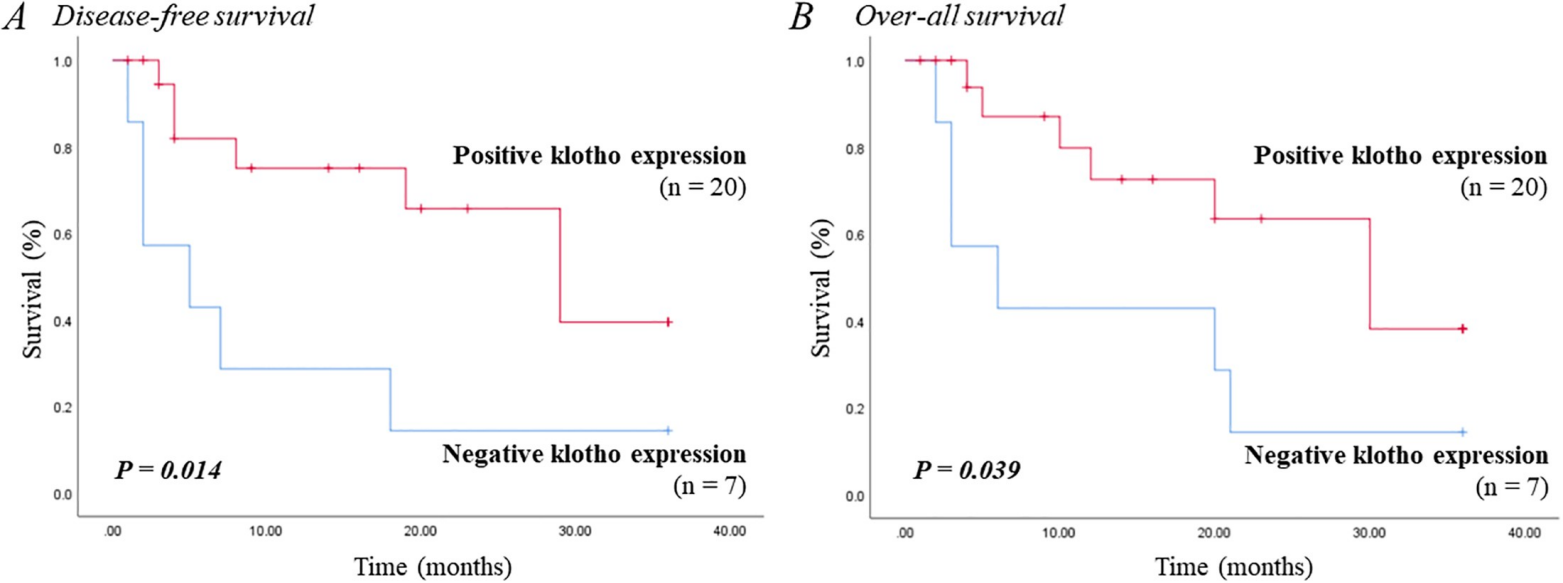
Kaplan–Meier survival curve of (A) disease-free survival and (B) overall survival based on the klotho expression of 27 dogs with malignant mammary gland tumor (median disease-free survival, 19 months; median overall survival, 21 months).

The disease-free and overall survival curves of 27 dogs with malignant mammary gland tumors were analyzed using Kaplan–Meier analysis. A total of 18 dogs died during the study period, of which 13 dogs died due to mammary gland tumors. The remaining nine dogs no longer visited the hospital or were alive during the study. Seven dogs showed negative klotho expression, whereas 20 showed positive expression. The analysis of the survival curves showed statistically significant association between negative klotho expression and poor disease-free survival. Poor overall survival was also associated with negative klotho expression. The median disease-free survival was 19 months, and the median overall survival was 21 months (Fig 2).

## Discussion

The present study investigated whether klotho is expressed within normal canine mammary glands and whether the expression changes in benign and malignant tumors. Immunohistochemistry confirmed klotho expression in canine mammary glands. A previous study has shown the expression of klotho protein in the kidney, reproductive organs, and brain of mice [29]. In human tissues, klotho expression was identified by immunohistochemistry in arterial, epithelial, endocrine, reproductive, and neuronal tissues [30]. Klotho has also been identified in normal human breast tissues. Although the exact physiological function of klotho in mammary glands has not yet been revealed, in addition to its function as a modulator of IGF-1 and FGF, klotho’s ability to activate calcium channels is believed to regulate the calcium concentration in milk [6]. It is the first study to observe klotho expression in canine mammary glands. Immunostaining for klotho was mainly seen in the cytoplasm and plasma membrane of epithelial cells. This is supported by studies showing that klotho is also a transmembrane protein [1] but is expressed in the early endosome, Golgi apparatus, and endoplasmic reticulum by binding to Na^+^/K^+^-ATPase and forming klotho and Na^+^, K^+^–ATPase complexes [31].

This is also the first study to study klotho expression in canine mammary gland tumors. It was confirmed that klotho expression was decreased in benign mammary gland tumors compared with normal mammary gland tissues and was further decreased in malignant tumors. Immunohistochemistry analysis of klotho expression in human breast cancer has confirmed significantly reduced staining in malignant breast cancers compared with normal tissues [6]. Studies on mammary hyperplasia also showed down-expression in tissues with atypical ductal hyperplasia and moderate hyperplasia, as compared to normal tissues and tissues with mild hyperplasia [32]. As benign mammary tumors are known to gradually progress into malignant tumors in dogs, it is not unusual for klotho expression to decrease as malignancy increases. In human breast cancer, klotho is known to suppress tumor growth by inhibiting the IGF-1 pathway and increasing the activation of the FGF pathway [6]. Another study has also confirmed the methylation of the klotho promoter in breast cancer cells and suggested that it may be an early mechanism of tumor development [32]. A similar mechanism is expected in canine mammary gland tumors, but further research is needed.

Histologic grade, Ki-67 level, and metastasis, which are relevant to clinical prognosis, have also been identified to be significantly positively associated with the absence of klotho expression. Negative klotho expression was also correlated with poor disease-free and overall survival. Human studies on oesophageal, ovarian cancer, and melanoma have shown that positive klotho expression is associated with improved survival and low metastatic rate [9, 13, 21]. The results of this study suggest the possibility of using negative klotho expression as a valuable prognostic factor in dogs with mammary gland tumors.

Focusing on the relationship between klotho and cancer, research is underway to develop a cancer treatment based on klotho. When soluble klotho was applied to the breast cancer cells, the activation of insulin and IGF-1 pathway, resulting in reduced proliferation, was identified [6]. Davidsohn et al. [22] investigated an *in vivo* study of three genes—namely, klotho, fibroblast growth factor 21, and a soluble form of the mouse transforming growth factor receptor 2—using an adeno-associated virus and showed that aging-related diseases, including heart failure, obesity, kidney failure, and diabetes, could be improved. This suggests that klotho can be applied through gene therapy methods. As klotho promoter methylation has been confirmed in cancer cells [28], Wang et al. [7] suggested the possibility of developing cancer treatment using klotho promoter demethylation with 5-aza-2′-deoxycytidine, resulting in an increased klotho expression. Nevertheless, this study is the first to confirm klotho expression in tumors in dogs. Further studies on the klotho mechanism in canine tumors are needed.

This study had several limitations. Beagles, from which normal mammary gland tissues were collected, were all 2 years old, which is relatively younger than patients with mammary gland tumors. Since *klotho* is a gene related to anti-aging, it is believed that there may be an age-related effect, but it was difficult to obtain tissues from old normal dogs. Moreover, among the dogs participating in this study, the median ages of the positive and negative klotho groups did not differ statistically significantly (age range: 6–15 years). In addition, this study did not include real-time polymerase chain reaction or western blotting to confirm mRNA or protein levels. The number of canine mammary gland tumor samples was relatively small. Further research is needed to address these points and to determine the klotho function in the canine mammary glands and mammary gland tumors.

In conclusion, this study provided evidence of the presence of klotho in canine mammary glands by immunohistochemistry. We confirmed the down-expression of klotho in canine mammary gland tumors, which was greater in malignant tumors than in benign tumors. The absence of klotho expression was significantly associated with tumor grade, Ki-67 expression, and metastasis. The negative klotho expression group had poor disease-free and overall survival. This result suggests that klotho may act as a diagnostic and prognostic marker.

## Supporting information

S1 Table Clinicopathological variables and points of immunohistochemistry for each dog used in this study. (DOCX)
