# Supplementary material for: Down-expression of klotho in canine mammary gland tumors and its prognostic significance
Source: PLoS One. 2022 Jun 6;17(6):e0265248. doi: 10.1371/journal.pone.0265248 (PMC9170104; doi:10.1371/journal.pone.0265248)
Supplement: S1 Table — (DOCX) [file pone.0265248.s001.docx]

S1 Table. Clinicopathological variables and points of immunohistochemistry for each dog used in this study.

|  | **Species** | **Sex** | **Age** | **Tumor size** | **Histopathology** | **Grade** | **IHC -klotho** | **IHC -klotho** | **IHC -ki67** | **IHC -ki67** | **Metastasis** | **Survival time** | **Disease free interval** |
| --- | --- | --- | --- | --- | --- | --- | --- | --- | --- | --- | --- | --- | --- |
| Normal 1 | Beagle | Female | 2 | - | - | - | 4.4 | High | - | - | - | - | - |
| Normal 2 | Beagle | Female | 2 | - | - | - | 3.6 | Low | - | - | - | - | - |
| Normal 3 | Beagle | Female | 2 | - | - | - | 7.8 | High | - | - | - | - | - |
| Normal 4 | Beagle | Female | 2 | - | - | - | 3.8 | Low | - | - | - | - | - |
| Normal 5 | Beagle | Female | 2 | - | - | - | 4.8 | High | - | - | - | - | - |
| Normal 6 | Beagle | Female | 2 | - | - | - | 7.8 | High | - | - | - | - | - |
| Normal 7 | Beagle | Female | 2 | - | - | - | 5.8 | High | - | - | - | - | - |
| Normal 8 | Beagle | Female | 2 | - | - | - | 10.8 | High | - | - | - | - | - |
| Benign 1 | Chihuahua | Female | 12 | ≤3 cm | Adenoma, simple | - | 3.8 | Low | - | - | - | - | - |
| Benign 2 | Yorkshire Terrier | Female | 13 | ≤3 cm | Adenoma, simple | - | 2.6 | Low | - | - | - | - | - |
| Benign 3 | Yorkshire Terrier | Female | 10 | ≤3 cm | Adenoma, simple | - | 3.2 | Low | - | - | - | - | - |
| Benign 4 | Yorkshire Terrier | Spayed female | 12 | ≤3 cm | Benign mixed tumor | - | 7.2 | High | - | - | - | - | - |
| Benign 5 | Cocker spaniel | Female | 6 | ≤3 cm | Adenoma, simple | - | 12.2 | High | - | - | - | - | - |
| Benign 6 | Mixed | Female | 8 | ≤3 cm | Adenoma, simple | - | 6.6 | High | - | - | - | - | - |
| Benign 7 | Toy Poodle | Female | 11 | ≤3 cm | Adenoma, complex | - | 10 | High | - | - | - | - | - |
| Benign 8 | Yorkshire Terrier | Spayed female | 16 | ≤3 cm | Benign mixed tumor | - | 3.6 | Low | - | - | - | - | - |
| Benign 9 | Yorkshire Terrier | Female | 14 | ≤3 cm | Adenoma, complex | - | 5.4 | High | - | - | - | - | - |
| Benign 10 | Yorkshire Terrier | Female | 15 | ≤3 cm | Adenoma, complex | - | 4 | Low | - | - | - | - | - |
| Benign 11 | Maltese | Spayed female | 13 | >3 cm | Adenoma, complex | - | 7.2 | High | - | - | - | - | - |
| Benign 12 | Cocker spaniel | Female | 7 | ≤3 cm | Adenoma, complex | - | 11.2 | High | - | - | - | - | - |
| Benign 13 | Mixed | Spayed female | 10 | ≤3 cm | Adenoma, complex | - | 8.6 | High | - | - | - | - | - |
| Benign 14 | Maltese | Female | 10 | ≤3 cm | Adenoma, complex | - | 7.2 | High | - | - | - | - | - |
| Benign 15 | Toy Poodle | Female | 13 | ≤3 cm | Benign mixed tumor | - | 8.6 | High | - | - | - | - | - |
| Benign 16 | Maltese | Female | 14 | ≤3 cm | Benign mixed tumor | - | 6.8 | High | - | - | - | - | - |
| Benign 17 | Yorkshire Terrier | Female | 8 | ≤3 cm | Adenoma, complex | - | 7.8 | High | - | - | - | - | - |
| Benign 18 | Mixed | Female | 16 | ≤3 cm | Benign mixed tumor | - | 11.4 | High | - | - | - | - | - |
| Benign 19 | Maltese | Female | 8 | ≤3 cm | Adenoma, complex | - | 10.6 | High | - | - | - | - | - |
| Benign 20 | Maltese | Female | 14 | ≤3 cm | Benign mixed tumor | - | 0 | Absent | - | - | - | - | - |
| Benign 21 | Yorkshire Terrier | Female | 14 | ≤3 cm | Adenoma, simple | - | 0 | Absent | - | - | - | - | - |
| Benign 22 | Maltese | Female | 8 | ≤3 cm | Adenoma, complex | - | 5 | High | - | - | - | - | - |
| Benign 23 | Maltese | Female | 11 | ≤3 cm | Adenoma, simple | - | 3.6 | Low | - | - | - | - | - |
| Benign 24 | Mixed | Female | 10 | ≤3 cm | Benign mixed tumor | - | 0 | Absent | - | - | - | - | - |
| Benign 25 | Maltese | Female | 8 | ≤3 cm | Adenoma, complex | - | 3.2 | Low | - | - | - | - | - |
| Benign 26 | Mixed | Spayed female | 11 | >3 cm | Adenoma, complex | - | 10 | High | - | - | - | - | - |
| Benign 27 | Maltese | Female | 12 | ≤3 cm | Adenoma, complex | - | 2.6 | Low | - | - | - | - | - |
| Benign 28 | Maltese | Female | 9 | ≤3 cm | Adenoma, complex | - | 2.8 | Low | - | - | - | - | - |
| Malignant 1 | Chihuahua | Female | 14 | >3 cm | Carcinoma, simple | 1 | 7.2 | High | 19.3 | Low | Yes | 12 | 4 |
| Malignant 2 | Yorkshire Terrier | Female | 13 | >3 cm | Carcinoma, complex | 2 | 3 | Low | 15.9 | Low | No | 10 | 8 |
| Malignant 3 | Maltese | Spayed female | 10 | >3 cm | Carcinoma, complex | 3 | 0 | Absent | 19.4 | High | Yes | 6 | 5 |
| Malignant 4 | Maltese | Spayed female | 7 | ≤3 cm | Carcinoma, complex | 1 | 7.4 | High | 11.7 | Low | No | 14+ | 14+ |
| Malignant 5 | Mixed | Female | Unknown | >3 cm | Carcinoma, complex | 2 | 2 | Low | 8.9 | Low | Yes | 20 | 19 |
| Malignant 6 | Yorkshire Terrier | Female | 9 | ≤3 cm | Carcinoma, simple | 1 | 6.6 | High | 13 | Low | No | 36+ | 36+ |
| Malignant 7 | Jindo | Spayed female | 13 | >3 cm | Carcinoma, mixed | 1 | 4.8 | High | 13.2 | Low | Yes | 30 | 29 |
| Malignant 8 | Toy Poodle | Female | 9 | >3 cm | Carcinoma, complex | 1 | 5.6 | High | 5.3 | Low | No | 36+ | 36+ |
| Malignant 9 | Shih-tzu | Female | 13 | ≤3 cm | Carcinoma, mixed | 3 | 3 | Low | 24.8 | High | Yes | 5 | 4 |
| Malignant 10 | Maltese | Female | 10 | >3 cm | Carcinoma, mixed | 1 | 8.2 | High | 7.5 | Low | No | 20+ | 20+ |
| Malignant 11 | Yorkshire Terrier | Female | 15 | ≤3 cm | Carcinoma, complex | 2 | 0 | Absent | 26.1 | High | Yes | 2 | 1 |
| Malignant 12 | Maltese | Spayed female | 12 | >3 cm | Carcinoma, complex | 3 | 0 | Absent | 19.2 | High | Yes | 20 | 7 |
| Malignant 13 | Mixed | Female | 15 | >3 cm | Adenosquamous carcinoma | 2 | 2.4 | Low | 19.5 | High | No | 36+ | 36+ |
| Malignant 14 | Shih-tzu | Female | 15 | >3 cm | Carcinoma, simple | 2 | 2 | Low | 27.7 | High | No | 3+ | 3+ |
| Malignant 15 | Toy Poodle | Female | 10 | >3 cm | Carcinoma, simple | 3 | 2.8 | Low | 14 | Low | Yes | 4 | 3 |
| Malignant 16 | Maltese | Spayed female | 11 | >3 cm | Carcinoma, complex | 1 | 0 | Absent | 16.2 | High | No | 36+ | 36+ |
| Malignant 17 | Dachshund | Female | 14 | >3 cm | Carcinoma, complex | 1 | 2 | Low | 19 | Low | No | 30 | 29 |
| Malignant 18 | Toy Poodle | Female | 12 | >3 cm | Carcinoma, mixed | 1 | 3 | Low | 13.5 | Low | No | 23+ | 23+ |
| Malignant 19 | Dachshund | Spayed female | 12 | ≤3 cm | Carcinoma, simple | 2 | 3.4 | Low | 42.6 | High | No | 4+ | 4+ |
| Malignant 20 | Toy Poodle | Spayed female | 14 | >3 cm | Carcinoma, simple | 3 | 0 | Absent | 24 | High | Yes | 21 | 18 |
| Malignant 21 | Cocker spaniel | Spayed female | 10 | ≤3 cm | Carcinoma, simple | 3 | 0 | Absent | 51.1 | High | Yes | 3 | 2 |
| Malignant 22 | Cocker spaniel | Spayed female | 10 | >3 cm | Carcinoma, simple | 3 | 0 | Absent | 6 | Low | Yes | 3 | 2 |
| Malignant 23 | Shih-tzu | Spayed female | 15 | >3 cm | Carcinoma, simple | 2 | 2.4 | Low | 5 | Low | No | 1+ | 1+ |
| Malignant 24 | Japanese Spitz | Spayed female | 9 | ≤3 cm | Carcinoma, simple | 1 | 3.6 | Low | 22.9 | High | No | 16+ | 16+ |
| Malignant 25 | Shih-tzu | Female | 11 | >3 cm | Carcinoma, simple | 1 | 3.4 | Low | 8 | Low | No | 9+ | 9+ |
| Malignant 26 | Mixed | Female | 8 | >3 cm | Carcinoma, simple | 2 | 4 | Low | 13.7 | Low | No | 3+ | 3+ |
| Malignant 27 | Toy Poodle | Spayed female | 13 | ≤3 cm | Carcinoma, simple | 3 | 4.4 | High | 28.4 | High | No | 2+ | 2+ |
|  |  |  |  |  |  |  |  |  |  |  |  |  |  |

+, indicating the dogs lost to follow up after that period
